# Early Prediction of Gestational Diabetes Mellitus and Insulin Therapy Requirement Using First-Trimester PAPP-A and Free β-hCG MoMs Levels: A Retrospective Case–Control Study

**DOI:** 10.3390/jcm13247725

**Published:** 2024-12-18

**Authors:** Gülay Balkaş, Şevki Çelen

**Affiliations:** Department of Perinatology, University of Health Sciences Etlik Zübeyde Women’s Health Care Training and Research Hospital, 06010 Ankara, Turkey; sevkicelen@yahoo.com

**Keywords:** pregnancy-associated plasma protein-A, free β-human chorionic gonadotropin, gestational diabetes mellitus, insulin-dependent gestational diabetes mellitus

## Abstract

**Objectives:** To evaluate the association between gestational diabetes mellitus (GDM), including insulin-dependent GDM with pregnancy-associated plasma protein-A (PAPP-A) multiples of the median (MoM) and free beta human chorionic gonadotropin (free β-hCG) MoM levels, and to assess their potential as predictive risk factors. **Methods:** This retrospective study included 2588 women with singleton pregnancies who underwent combined first-trimester screening, along with the 50 g glucose challenge test (GCT) and a 100 g oral glucose tolerance test (OGTT) between 24 and 28 weeks of gestation. Patients were initially divided into four groups based on the glucose screening results, and PAPP-A and free β-hCG MoMs were compared between these groups. GDM cases managed by diet were then compared with those requiring insulin therapy. **Results:** Of the study population, 132 women (5.10%) were diagnosed with GDM, 112 (84.8%) managed their glycemia with dietary changes, while 20 (15.2%) required insulin therapy. PAPP-A levels were significantly lower in the GDM group compared to the control group (*p* < 0.001). In addition, the insulin-dependent GDM group had significantly lower PAPP-A levels than the diet-controlled group (*p* < 0.001). No significant differences were observed in the free β-hCG MoM levels between the groups (*p* = 0.292). Receiver operating characteristic analysis identified 0.815 as the optimal PAPP-A cut-off value for predicting GDM, with a sensitivity of 61.4%, specificity of 61.6%, and an area under the curve (AUC) of 0.649 (95% CI: 0.595–0.703). For insulin-dependent GDM, the same threshold yielded an AUC of 0.621 (95% CI: 0.563–0.679), with a sensitivity of 58.6% and a specificity of 59.7%. **Conclusions:** Low serum PAPP-A MoM levels are significantly associated with the development of GDM, including insulin-dependent cases. Although PAPP-A alone may not be a definitive predictive marker for GDM, low levels could support the recommendation for early screening as part of a broader diagnostic approach.

## 1. Introduction

Gestational diabetes mellitus (GDM) is the most common metabolic disorder of pregnancy, typically diagnosed in the late second trimester [1] and is associated with significant adverse maternal and fetal outcomes [2]. Between 2011 and 2017, the prevalence of GDM increased by 9%, driven by factors such as reduced physical activity, the global obesity epidemic, and rising maternal age at pregnancy [3]. The prevalence of GDM varies by ethnicity, geographical region, and diagnostic criteria [4], with estimates in Turkey at 10.9% and a global range of 4% to 16% [5].

Increased maternal and fetal risks are correlated with the severity and duration of maternal hyperglycemia [6], leading to complications such as fetal anomalies, spontaneous abortion, stillbirth, pre-eclampsia, polyhydramnios, neonatal respiratory distress syndrome, and metabolic disorders like neonatal hypoglycemia, hyperbilirubinemia, and hypocalcemia [1]. In addition, GDM is associated with long-term risks, including type 2 diabetes, metabolic syndrome, and future cardiovascular disease [7,8].

Physiologically, maternal insulin resistance increases significantly from the 24th week of gestation to meet fetal nutritional needs, but in women with inadequate insulin secretion, this may result in hyperglycemia [9]. As a result, glucose screening for GDM is routinely performed between 24 and 28 weeks of gestation in all pregnant women, regardless of defined risk factors [10]. Studies have shown that elevated first-trimester fasting glucose levels, even within the non-diabetic range, are associated with an increased risk of GDM and adverse pregnancy outcomes [11], suggesting that metabolic changes associated with GDM may manifest early in pregnancy.

Consequently, first-trimester biomarkers may offer opportunities for early diagnosis and treatment, potentially improving maternal and fetal outcomes. Abnormal levels of maternal serum biomarkers, used in the first-trimester screening, have been associated with adverse maternal and perinatal outcomes [12,13]. Low levels of pregnancy-associated plasma protein-A (PAPP-A) and free beta human chorionic gonadotropin (free β-hCG), in the absence of aneuploidy, have been associated with an increased risk of preterm birth, pre-eclampsia, and low birth weight [12,14,15]. Recently, attention has focused on these biomarkers as potential predictors of GDM [16]. While some studies have suggested that low levels of PAPP-A and free β-hCG are associated with an increased risk of GDM [17,18,19], others have reported no significant association [20,21,22].

Despite the fact that 80% of the global burden of diabetes occurs in low- and middle-income countries like Turkey, a significant number of pregnant women in these regions do not undergo screening for GDM [23]. In Turkey, many pregnant women decline the recommended GDM screening tests due to concerns that consuming the glucose solution may increase the risk of diabetes in their infants. This gap in screening is particularly concerning given the absence of reliable and accessible biomarkers for the early detection of GDM. Given the high prevalence of GDM among Turkish women and the critical importance of early diagnosis, the development of efficient and cost-effective screening methods is essential. Interestingly, many women in these regions do undergo the first- trimester aneuploidy screening test due to concerns about potential disabilities in their offspring. 

Therefore, the aim of this study was to investigate the association of PAPP-A MoM and free β-hCG MoM levels with the development of GDM, including insulin-dependent GDM, and to assess their potential as predictive markers. First-trimester screening provides multiples of the median (MoM) values for these markers, which are particularly valuable for practitioners in peripheral healthcare settings. Unlike previous studies conducted in Turkey, which focused on mean PAPP-A concentrations, this study aimed to report PAPP-A MoM and free β-hCG MoM values, providing a more standardized approach to result interpretation.

## 2. Materials and Methods

### 2.1. Study Design and Eligibility Criteria

This retrospective case–control study included patients aged 18 to 45 years with a singleton pregnancy who underwent antenatal assessments and delivered at a tertiary care center between January 2020 and December 2022. Antenatal assessments involved first-trimester aneuploidy screening using ultrasound and maternal serum biomarkers, including PAPP-A and free β-hCG, followed by GDM screening in the late second trimester. Patients with fetal abnormalities, multiple gestations, and pregestational diseases such as nephropathy, pregestational diabetes, thyroid dysfunction, and hypertension were excluded from the study.

### 2.2. Data Collection

Patient data were obtained from the hospital’s electronic database and medical records. The collected data encompassed patient age, body mass index (BMI), gestational age at the time of testing, maternal weight, crown–rump length (CRL), and nuchal translucency (NT) measurements from the first-trimester ultrasound. First-trimester screening was performed using the GE Healthcare Voluson E6 Expert ultrasound machine equipped with a 3–5 MHz convex/broadband transducer, following the Fetal Medicine Foundation protocols for measuring CRL and NT [24]. Gestational age was determined based on the last menstrual period and confirmed by ultrasound. Maternal serum biomarkers, free β-hCG, and PAPP-A levels were measured using an automated instrument (IMMULITE 2000) as part of the routine first-trimester antenatal screening for aneuploidy between 11^+0^ and 13^+6^ weeks’ gestation. The results were converted to the multiples of the median (MOM) and adjusted for gestational age, maternal weight, and smoking status. 

### 2.3. Diagnosis of Gestational Diabetes Mellitus

All patients underwent routine GDM screening between 24 and 28 weeks’ gestation. The initial screening included a 50 g glucose challenge test (GCT), which was performed regardless of the time of day or the patient’s last meal. A result of 140 mg/dL or higher on the GCT required a follow-up oral 100 g glucose tolerance test (OGTT) after an overnight fast of 8 to 12 h. The diagnosis of GDM was made according to the criteria of the American College of Obstetricians and Gynecologists [25], requiring at least two of the following glucose levels to be exceeded for a positive diagnosis: fasting, 95 mg/dL; 1 h, 180 mg/dL; 2 h, 155 mg/dL; and 3 h, 140 mg/dL. In addition, a 50 g GCT result of 200 mg/dL or higher was also considered diagnostic for GDM.

### 2.4. Statistical Analysis

Data analysis was performed using SPSS software (version 26.0, SPSS Inc., Chicago, IL, USA). Qualitative variables were presented as frequency distributions, while normally distributed quantitative data were expressed as mean (standard deviation (SD)) and non-normally distributed data as median (interquartile range (IQR)). Group comparisons were made using either an independent t-test or one-way ANOVA for normally distributed data, and the chi-square test or Fisher’s exact test for categorical variables. A one-way analysis of covariance (ANCOVA) was conducted to compare PAPP-A measurements across groups, with BMI included as a covariate to control for its potential confounding effect. Following adjustment for BMI, Bonferroni post-hoc analysis was applied to determine the sources of observed group differences. Logistic regression analysis was used to determine the relative risk of the association between PAPP-A levels and both GDM and insulin-dependent GDM. In addition, receiver operating characteristic (ROC) curve analysis and the area under the curve (AUC) were used to predict both GDM and insulin-dependent GDM. Differences were considered significant if the *p* value was <0.05. 

The study was conducted in accordance with the principles of the Declaration of Helsinki and received ethical approval from the Ethics Committee of the University of Health Sciences, Etlik Zubeyde Women’s Health Care Training and Research Hospital (No: 20.05.2021-07). Due to the retrospective nature of this study, informed consent was waived with the approval of the local ethics committee.

## 3. Results 

Patients with a singleton pregnancy attending our outpatient clinic for first-trimester genetic screening, along with the 50 g GCT and 100 g OGTT between 24 and 28 weeks’ gestation, were recruited. Detailed maternal and fetal characteristics of the study population are shown in Table 1. The main characteristics included a median maternal age of 30 years (interquartile range (IQR): 19–46 years), a median gravidity of 2 (IQR: 1–10), and a median BMI of 29 (IQR: 18–43). 

First-trimester screening was performed at a median gestational age of 12 weeks (IQR: 11–14 weeks) with median CRL and NT measurements of 63.5 mm (IQR: 50–81 mm) and 1.1 mm (IQR: 0.9–1.4 mm), respectively. The median gestational age at delivery was 38 weeks (IQR: 32–41 weeks) and the median birth weight was 3247 g (IQR: 2000–4690 g). 

The categorization of the patients is shown in Table 2. Of the 2588 pregnant women enrolled in the study, 2260 with 50 g GCT results below 140 mg/dL were assigned to the control group (group a). Those with 50 g GCT results of 140 mg/dL or higher were further categorized based on their 100 g OGTT results. Group b consisted of 137 patients with normal glucose levels at all four time points during the 100 g OGTT. Group c consisted of 132 patients diagnosed with GDM. Finally, group d consisted of 59 patients with a single elevated glucose value after the 100 g OGTT who were classified as having impaired glucose tolerance (IGT).

No significant differences were observed between the groups in terms of NT and CRL measurements (*p* > 0.05). However, the GDM group had a significantly higher mean age (33.9 ± 5.5 years), BMI (31.1 ± 4.4), and gravidity rate (3.1 ± 1.7) compared to the other groups (*p* < 0.001). In addition, a significant difference in PAPP-A MoM values was found between the control group (1.2 ± 0.7) and both the IGT (1.0 ± 0.7) and GDM (0.8 ± 0.5) groups (*p* < 0.001). The free β-hCG MoM levels were not significantly different between the groups (*p* = 0.292). 

As shown in Table 2, there was a significant difference in BMI between groups a, b, c, and d, with a progressively increasing BMI associated with OGTT response. Given that GDM is multifactorial, with metabolic status and body fat playing an important role in insulin resistance, IGT, and GDM, a one-way analysis of covariance (ANCOVA) was performed to compare PAPP-A MoM between groups, with BMI included as a covariate to account for its potential confounding effect (Table 3). After adjustment for BMI, a significant difference in PAPP-A levels was observed between the groups (*p* < 0.001). A Bonferroni post-hoc analysis was then performed to identify the source of this difference. The results showed that the adjusted mean PAPP-A levels were significantly higher in the control group than in the GDM group.

The overall prevalence of GDM in this study population was 5.10% (n = 132) cases. As shown in Table 4, 112 women (84.8%) with GDM managed their glycemia with dietary changes, while 20 women (15.2%) required insulin therapy. PAPP-A MoM was significantly lower in the insulin-dependent GDM group compared to the diet-controlled group (0.9 ± 0.5 vs. 1.1 ± 0.7; *p* < 0.001). No significant difference was observed in free β-hCG MoM levels between these groups (*p* = 0.294). 

**Figure 1:**
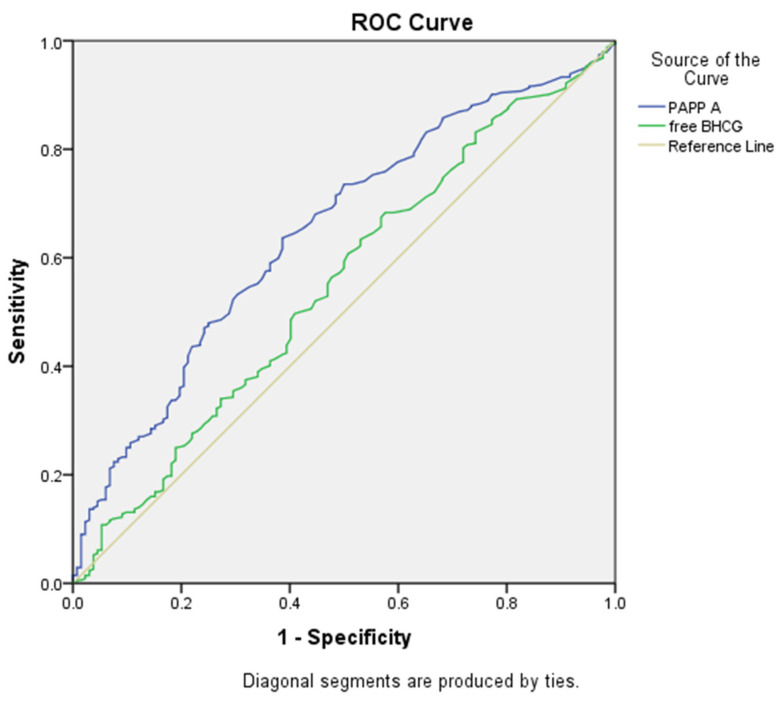
Receiver operating characteristic (ROC) analysis showed that the optimal cut-off point for PAPP-A MoM in predicting the development of gestational diabetes was 0.815, yielding a sensitivity of 61.4% and a specificity of 61.6%, with an area under the curve (AUC) of 0.649 (*p* < 0.001). ROC curve of free β-hCG, cut-off value 0.965, AUC 0.55, sensitivity of 53%, and a specificity of 55.2% (*p* = 0.091).

**Figure 2:**
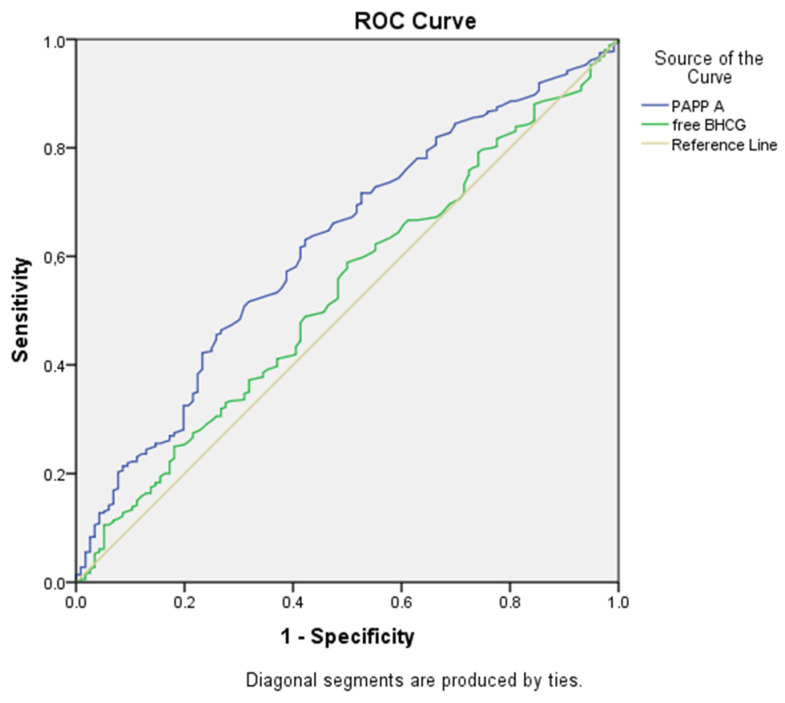
Receiver operating characteristic (ROC) analysis for PAPP-A MoM and free β-hCG MoM for the prediction of insulin-dependent GDM in the first trimester. PAPP-A MoM in predicting insulin-dependent gestational diabetes was 0.815, yielding a sensitivity of 58.6% and a specificity of 59.7%, with an AUC of 0.379 (*p* < 0.001). Free β-hCG ROC curve analysis showed a cut-off of 0.985, an AUC of 0.532, a sensitivity of 51.7%, and a specificity of 52.2% (*p* = 0.292).

The performance of PAPP-A MoM in the prediction of both GDM and insulin-dependent GDM was analyzed using the ROC curve. Logistic regression analysis showed a significant association between PAPP-A MoM and both GDM and insulin-dependent GDM (*p* < 0.05). As shown in Table 5, a decrease in PAPP-A MoM was associated with an increased risk of GDM (1/0.491) and an increased risk of insulin-dependent GDM (1/0.394). Subsequent ROC analysis determined that the optimal cut-off value for PAPP-A MoM to predict GDM was 0.815, yielding a sensitivity of 61.4% and a specificity of 61.6%, with an AUC of 0.649 (95% CI 0.595–0.703) (Figure 1). For insulin-dependent GDM, the ROC analysis yielded an AUC of 0.621 (95% CI: 0.563–0.679) at the same cut-off point, with sensitivity and specificity values of 58.6% and 59.7%, respectively (Figure 2). 

## 4. Discussion 

GDM is a prevalent pregnancy complication that, if not properly managed, can lead to adverse maternal and fetal outcomes. Early detection and timely intervention are crucial to achieving optimal outcomes. This study investigated the potential of first-trimester biomarkers, PAPP-A MoM and free β-hCG MoM levels, which are routinely used for aneuploidy screening between 11 and 14 weeks’ gestation, as possible screening markers for GDM, including insulin-dependent GDM. 

The present study demonstrated a significant association between PAPP-A MoM levels and GDM, with patients who developed GDM (0.8) exhibiting significantly lower levels compared to the control group (1.2) (*p* < 0.001). This finding is consistent with a retrospective study by Beneventi et al. [19], which reported a PAPP-A MoM of 0.7 in women with GDM, significantly lower than that of the control group (*p* < 0.001). Moreover, in a retrospective analysis of 2410 cases, Visconti et al. [26] identified an increased risk of GDM when PAPP-A MoM was less than one.

In our study, a significant difference in PAPP-A MoM levels was observed between the control group and both the IGT and GDM groups (*p* < 0.001). Similarly, in a retrospective study of 278 women, Caliskan et al. [27] divided the study population into four groups based on their 50 g and 100 g OGTT results and reported lower PAPP-A levels in the GDM group compared with the other groups (*p* < 0.01). The primary distinction between the two studies is that we reported PAPP-A MoM values, whereas they measured mean PAPP-A concentrations.

In a large study conducted by Ong et al. [28] involving 5584 singleton pregnancies, PAPP-A MoM values were categorized into percentiles, with values below the 10th percentile significantly associated with gestational diabetes (*p* = 0.002). Furthermore, a prospective study by Ramezani et al. [29], which included 284 pregnant women, showed that those with low PAPP-A levels had a 4.77-fold increased risk of developing GDM compared to those with normal PAPP-A levels. 

In a prospective study involving 1664 women, Wells et al. [30] classified GDM into early- and late-onset groups, based on diagnosis before or after 22 weeks’ gestation. They reported that PAPP-A MoM levels were reduced by 41.3% in women with type 2 diabetes, 22.6% in those with early-onset GDM, and 8.6% in those with late-onset GDM. In line with these findings, our study demonstrated a 16.6% decrease in PAPP-A MoM levels in the IGT group and a 33.3% decrease in the GDM group compared with the control group. 

However, several studies have reported no significant association between PAPP-A levels and the development of GDM [20,21,22]. The multifactorial nature of GDM, along with variations in diagnostic criteria, study design, and differences in population characteristics—such as ethnicity—may influence the severity of GDM and could account for the inconsistent findings observed in previous research. 

Previous research has investigated whether low PAPP-A levels in the first trimester serve as an independent risk factor for GDM, with conflicting results. A meta-analysis of 17 studies conducted by Talasaz et al. [31] suggested that low PAPP-A levels in early pregnancy were associated with GDM, reporting a sensitivity of 55% (53–58%) and specificity of 90% (89–90%), with a positive likelihood ratio of 2.48 (0.83–7.36) and negative likelihood ratio of 0.70 (0.45–1.09). Similarly, a meta-analysis by Lovati et al. [32] found an AUC-ROC of 0.70 (95% CI: 0.60–0.74), supporting the predictive ability of PAPP-A for GDM. In addition, Spencer et al. [17] identified PAPP-A as a weak but statistically significant predictor of GDM, with an AUC-ROC of 0.55 (95% CI: 0.53–0.57). 

In contrast, a prospective observational study of 31,225 singleton pregnancies (of which 787 developed GDM) by Syngelaki et al. [18] found a lower median PAPP-A MoM (0.949) in the GDM group compared with the unaffected group (*p* = 0.0009). However, they concluded that PAPP-A levels were not effective for GDM screening, and that incorporating PAPP-A did not improve screening performance based on maternal factors. Similarly, studies by Savvidou et al. [20] and Cheuk et al. [22] found no significant association between low PAPP-A levels and GDM. 

In our study, the cut-off point for PAPP-A MoM in predicting GDM was determined to be 0.815, with a sensitivity of 61.4% and a specificity of 61.6%. PAPP-A was identified as a weak but significant independent risk factor for GDM, with an AUC-ROC of 0.649 (95% CI 0.595–0.703). 

The varying severity of GDM in the study populations may account for the discrepancies between studies. For instance, in the studies conducted by Spencer et al. [17] and Beneventi et al. [19], the percentages of women with GDM requiring insulin treatment were 12% and 23.2%, respectively. In contrast, Cheuk et al. [22] reported that only two women (1.2%) with GDM required insulin therapy. 

Syngelaki et al. [18] further stratified their study population into three groups based on GDM treatment: diet-controlled (36.7%), metformin (18.2%), and insulin (46.1%). They found that the PAPP-A levels varied between these groups, with the lowest levels observed in the insulin-treated GDM group (0.896) and the highest in the diet-controlled group (1.0155) (*p* < 0.05). Consistent with Syngelaki et al. [18], Lovati et al. [32] also reported that low PAPP-A MoM levels were associated with an increased risk of requiring insulin therapy in women with GDM (*p* < 0.001). In contrast, Husslein et al. [21], focusing exclusively on insulin-treated GDM patients, suggested that these women did not have altered PAPP-A levels at 11–14 weeks. In the present study, which included 20 patients (15.2%) requiring insulin therapy, the PAPP-A MoM levels were significantly lower in the insulin-dependent GDM group compared to the diet-controlled GDM group (*p* < 0.001). Using a cut-off value of 0.815 for PAPP-A MoM, the sensitivity for predicting insulin-dependent GDM was 58.6%, while the specificity was 59.7%.

The present study found no significant differences in free β-hCG MoM levels between women with GDM and other groups. This finding is consistent with previous studies that similarly reported no significant differences in serum free β-hCG levels between women with GDM and the controls [20,28,33,34]. In contrast, Spencer et al. [17] identified free β-hCG MoM as a weak but significant predictor of GDM, with an AUC-ROC of 0.54 (95% CI: 0.52–0.56). Likewise, a meta-analysis by Donovan et al. [35] found that women with GDM had lower levels of both PAPP-A and free β-hCG in the first trimester compared with the controls (*p* < 0.00001, *p* = 0.005, respectively). 

Although our study provides valuable insights into the early risk assessment of GDM, its limitations, including its retrospective design, must be acknowledged. Prospective studies are needed to validate these findings and further refine strategies for early detection of GDM.

## 5. Conclusions

The actual prevalence of GDM may be higher than observed, as a significant number of pregnant women decline the recommended screening test, often due to concerns that consuming the glucose solution may increase the risk of diabetes in their offspring. This reluctance may result in undiagnosed cases of GDM that remain unrecognized and untreated, highlighting the critical need to prioritize early and accurate diagnosis, antenatal care, and treatment strategies to minimize the risk of complications and adverse outcomes for both mothers and infants. Low serum PAPP-A MoM levels are significantly associated with the development of GDM, including insulin-requiring GDM. Although the predictive value of PAPP-A as an independent marker for GDM remains limited, its low levels could support the implementation of earlier screening protocols within a comprehensive diagnostic framework. Further research is needed to evaluate how PAPP-A can be combined with other clinical factors to improve the early detection of GDM and facilitate more personalized management plans for at-risk pregnancies, ultimately improving maternal and fetal health outcomes. 

## Figures and Tables

**Table 1:** Maternal and pregnancy characteristics of the study population.

Variables	Median (IQR)	Mean ± SD
PAPP-A (MoM)	0.89 (0.17–3.37)	1.04 ± 0.64
Free β-hCG (MoM)	1 (0.15–3.17)	1.1 ± 0.52
Age (years)	30 (19–46)	30.82 ± 6.13
BMI (kg/m^2^)	29 (18–43)	29.36 ± 4.54
Gravidity	2 (1–10)	2.68 ± 1.49
Parity	1 (1–6)	1.25 ± 1.06
Delivery week	38 (32–41)	38.08 ± 1.58
Birth weight (grams)	3247 (2000–4690)	3212.93 ± 442.33
Gestational age at the time of first-trimester screening (weeks)	12 (11–14)	11.4 ± 0.92
Gestational age at OGTT screening (weeks)	25 (24–26)	25 ± 0.8
CRL (mm)	60.5 (50–81)	63.5 ± 9.2

Abbreviations: PAPP-A: pregnancy-associated plasma protein-A; free β-hCG: free beta human chorionic gonadotropin; NT: nuchal translucency; CRL: crown–rump length; OGTT: oral glucose tolerance test; BMI: body mass index; SD: standard deviation; MoM: multiples of the median; IQR: inter quartile range.

**Table 2:** Comparison of maternal biomarkers and pregnancy characteristics between groups.

Patient Groups	n	PAPP-A (MoM)	Free β-hCG (MoM)	Maternal Age (Years)	BMI (kg/m^2^)	Gravida	Parity	Delivery (Weeks)	Birth Weight (Grams)
		(Mean ± SD)	(Mean ± SD)	(Mean ± SD)	(Mean ± SD)	(Mean ± SD)	(Mean ± SD)	(Mean ± SD)	(Mean ± SD)
Group a	2588	1.2 ± 0.7	1.2 ± 0.5	27.1 ± 4.6	28.4 ± 4.2	2.3 ± 1.4	1 ± 0.9	38.5 ± 1.4	3150.9 ± 423.5
Group b	137	1.1 ± 0.6	1.1 ± 0.5	31.1 ± 6	29.7 ± 4.5	2.7 ± 1.4	1.2 ± 1	38.4 ± 1.6	3230.4 ± 422.6
Group c	132	0.8 ± 0.5	1 ± 0.5	33.9 ± 5.5	31.1 ± 4.4	3.1 ± 1.7	1.5 ± 1.2	37.5 ± 1.6	3247.7 ± 483.2
Group d	59	1 ± 0.7	1 ± 0.5	32.6 ± 6.3	30.4 ± 5.1	2.6 ± 1.2	1.3 ± 1	37.7 ± 1.4	3250.2 ± 432.4
*p* *		<0.001	0.292	<0.001	<0.001	<0.001	0.004	< 0.001	0.226
		a > b, c, d & b > c		a < b, c, d & b < c	a < b, c, d & b < c	c > a, b, d	c > a, b, d	a, b > c, d	

Abbreviations: Group a: control; group b: only 50 g glucose challenge test is high; group c: gestational diabetes mellitus; group d: only one value in 100 g glucose tolerance test is high; PAPP-A: pregnancy-associated plasma protein-A; free β-hCG: free beta human chorionic gonadotropin; BMI: body mass index; MoM: multiples of the median; SD: standard deviation; * one-way ANOVA statistics; *p* < 0.05 compared with the control groups.

**Table 3:** BMI adjusted comparison of PAPP-A levels between groups.

Patient Groups	n	PAPP-A (MoM)
		(Adjusted Mean ± SD)
Group a	2588	1.2 ± 0.1
Group b	137	1.1 ± 0.1
Group c	132	0.9 ± 0.1
Group d	59	1 ± 0.1
*p* *		<0.001
		a, b, d > c

Abbreviations: PAPP-A: pregnancy-associated plasma protein-A; MoM: multiples of the median; BMI: body mass index; SD: standard deviation; * one-way analysis of covariance (ANCOVA) statistics was performed to compare PAPP-A MoM between groups, with BMI included as a covariate to account for its potential confounding effect.

**Table 4:** Clinical characteristics of the groups based on the management of GDM.

Patient Groups	n (132)	PAPP-A (MoM)	Free β-hCG (MoM)	Maternal Age (Years)	BMI (kg/m^2^)	Gravida	Parity	Delivery Week	Birth Weight (Grams)
		Mean ± SD	Mean ± SD	Mean ± SD	Mean ± SD	Mean ± SD	Mean ± SD	Mean ± SD	Mean ± SD
Diet- controlled GDM	112	1.1 ± 0.7	1.1 ± 0.5	29.8 ± 5.9	29.4 ± 4.5	2.5 ± 1.4	1.1 ± 1	38.3 ± 1.5	3220.4 ± 434.8
Insulin- dependent GDM	20	0.9 ± 0.5	1.1 ± 0.5	34 ± 5.6	30.9 ± 4.6	3.2 ± 1.6	1.6 ± 1.2	37.5 ± 1.7	3189.7 ± 466.1
*p* *		<0.001	0.294	<0.001	0.003	<0.001	<0.001	<0.001	0.517

Abbreviations: PAPP-A: pregnancy-associated plasma protein-A; free β-hCG: free beta human chorionic gonadotropin; MoM: multiples of the median; GDM: gestational diabetes mellitus; SD: standard deviation; * two-sample *t*-test; *p* < 0.05 compared with the control groups.

**Table 5:** Logistic regression analysis of the effect of PAPP-A on GDM and insulin-dependent GDM.

Variables	Groups	OR (%95 CI)	*p*
PAPP-A (MoM)	GDM	0.491 (0.322–0.748)	<0.001
PAPP-A (MoM)	Insulin-dependent GDM	0.394 (0.260–0.596)	<0.001

Abbreviations: PAPP-A: pregnancy-associated plasma protein-A; MoM: multiples of the median; GDM: gestational diabetes mellitus, CI: confidence interval.

## Data Availability

The data supporting the findings of this study are available from the corresponding author (GB) upon reasonable request.
